# A Real-World Study on the Clinical Characteristics, Outcomes, and Relationship between Antibiotic Exposure and *Clostridioides difficile* Infection

**DOI:** 10.3390/antibiotics13020144

**Published:** 2024-02-01

**Authors:** Bogdan Ioan Vintila, Anca Maria Arseniu, Claudiu Morgovan, Anca Butuca, Victoria Bîrluțiu, Carmen Maximiliana Dobrea, Luca Liviu Rus, Steliana Ghibu, Alina Simona Bereanu, Rares Arseniu, Ioana Roxana Codru, Mihai Sava, Felicia Gabriela Gligor

**Affiliations:** 1 Clinical Surgical Department, Faculty of Medicine, “Lucian Blaga” University of Sibiu, 550169 Sibiu, Romania; bogdan.vintila@ulbsibiu.ro (B.I.V.); ioanaroxana.bera@ulbsibiu.ro (I.R.C.); mihai.sava@ulbsibiu.ro (M.S.); 2 County Clinical Emergency Hospital, 550245 Sibiu, Romania; victoria.birlutiu@ulbsibiu.ro; 3 Preclinical Department, Faculty of Medicine, “Lucian Blaga” University of Sibiu, 550169 Sibiu, Romania; claudiu.morgovan@ulbsibiu.ro (C.M.); anca.butuca@ulbsibiu.ro (A.B.); carmen.dobrea@ulbsibiu.ro (C.M.D.); liviu.rus@ulbsibiu.ro (L.L.R.); felicia.gligor@ulbsibiu.ro (F.G.G.); 4 Clinical Medical Department, Faculty of Medicine, “Lucian Blaga” University of Sibiu, 550169 Sibiu, Romania; 5 Department of Pharmacology, Physiology and Pathophysiology, Faculty of Pharmacy, “Iuliu Hatieganu” University of Medicine and Pharmacy, 400012 Cluj-Napoca, Romania; steliana.ghibu@umfcluj.ro; 6 County Emergency Clinical Hospital ”Pius Brînzeu”, 300723 Timișoara, Romania; arseniurares@gmail.com

**Keywords:** *Clostridioides difficile*, *Clostridioides difficile* infections, CDI, antibiotic exposure, EudraVigilance, single-center retrospective study, healthcare-associated infections

## Abstract

*Clostridioides difficile* is a Gram-positive bacteria that causes nosocomial infections, significantly impacting public health. In the present study, we aimed to describe the clinical characteristics, outcomes, and relationship between antibiotic exposure and *Clostridioides difficile* infection (CDI) in patients based on reports from two databases. Thus, we conducted a retrospective study of patients diagnosed with CDI from Sibiu County Clinical Emergency Hospital (SCCEH), Romania, followed by a descriptive analysis based on spontaneous reports submitted to the EudraVigilance (EV) database. From 1 January to 31 December 2022, we included 111 hospitalized patients with CDI from SCCEH. Moreover, 249 individual case safety reports (ICSRs) from EVs were analyzed. According to the data collected from SCCEH, CDI was most frequently reported in patients aged 65–85 years (66.7%) and in females (55%). In total, 71.2% of all patients showed positive medical progress. Most cases were reported in the internal medicine (n = 30, 27%), general surgery (n = 26, 23.4%), and infectious disease (n = 22, 19.8%) departments. Patients were most frequently exposed to ceftriaxone (CFT) and meropenem (MER). Also, in the EV database, most CDI-related ADRs were reported for CFT, PIP/TAZ (piperacillin/tazobactam), MER, and CPX (ciprofloxacin). Understanding the association between previous antibiotic exposure and the risk of CDI may help update antibiotic stewardship protocols and reduce the incidence of CDI by lowering exposure to high-risk antibiotics.

## 1. Introduction

*Clostridioides difficile* (CD) is a bacterium characterized by its Gram-positive nature, spore-forming ability, and anaerobic properties [1,2]. The microorganism commonly occurs in the human gastrointestinal tract, which harbors a variety of bacteria, mainly anaerobic, but it can also occur in animals and various environments [3].

CD infection (CDI) is a highly prevalent hospital-acquired infection [4] that has increased in frequency and severity over the past decade. CD most often causes healthcare-associated infections. They are one of the top three threats to public health, according to the Centers for Disease Control and Prevention [5,6]. On the other hand, a recent population-based study found that as much as 41% of CDI cases are actually contracted in the community. Interestingly, the study also revealed that while community-acquired CDI generally has a milder clinical course than the hospital-acquired form, it is still a significant concern. It is essential to be aware of the prevalence of CDI in community settings to identify high-risk individuals early on [7].

CDI is frequently linked to a set of risk factors. Many factors contribute to CDI, including antimicrobial use, advanced age, hospitalization, and a compromised immune system. Advanced age is strongly associated with complications and death, especially in patients with co-infections and high comorbidity scores [8].

It is a well-known fact that patients who receive antibiotics during their hospital stay are at a higher risk of developing CDI [9,10]. Carbapenems are a class of broad-spectrum antibiotics that are highly effective against many bacteria, including Gram-positive and Gram-negative bacteria. They are considered last-resort antibiotics typically used to treat severe and often life-threatening infections resistant to other antibiotics [11]. However, their increasing use is a matter of concern for several reasons, and one is the imbalances produced in the microbiota, with a high risk of CDI [12]. A member of the oxazolidinone antibiotic family, linezolid (LIN) stands out as the first representative of its class [13]. The drug has been approved for treating infections caused by *Enterococcus faecium*, which is resistant to vancomycin; *Staphylococcus aureus* pneumonia, which occurs in hospitals; and complex skin and skin structure infections. Furthermore, LIN is well known for its effectiveness as an antibiotic in treating infections in the ICU [13]. In recent years, there has been renewed attention on polymyxins due to the emergence of Gram-negative bacteria resistant to multiple antibiotics, leaving few alternative treatment options available [14]. It is common for healthcare professionals to administer antibiotics, including piperacillin and tazobactam (PIP/TAZ), ceftriaxone (CFT), ciprofloxacin (CPX), and gentamicin (GEN), in intensive care settings. PIP/TAZ is recognized for its β-lactam/β-lactamase solid-inhibiting properties [15,16]. Upon conducting a thorough examination of the medical records of 640 patients treated in an ICU, it was discovered that a significant majority of 73.4% of patients had received CFT. Interestingly, it was noted that CPX and GEN were administered to fewer than 3% of patients who were admitted to the ICU [17]. One notable benefit of using CPX and GEN to treat patients who are critically ill is that they can effectively treat pathogens that are not as responsive to the typical antibiotics administered in intensive care scenarios, particularly in the context of urinary tract infections [18,19].

Healthcare providers can improve patient care in managing CDI by using a range of therapeutic techniques and expanding their knowledge of the unique characteristics of individuals at higher risks of acquiring infections during their hospital stay [20]. This information allows for tailoring antibiotic therapies and decreasing the probability of contracting hospital-acquired infections, such as CDI [21,22].

In the present study, we aimed to describe the clinical characteristics, outcomes, and relationship between previous antibiotic exposure and *Clostridioides difficile* infection in patients based on reports from two databases. We conducted a retrospective analysis of medical records and data for patients diagnosed with healthcare-associated CDI at the Sibiu County Clinical Emergency Hospital (SCCEH) in 2022. We analyzed patient demographics, comorbidity scores, antibiotic prescriptions, the duration of hospitalization, the need for intensive care admission, and clinical outcomes related to *Clostridioides difficile* infection. The antibiotics of interest for our study were PIP/TAZ, CFT, CPX, GEN, meropenem (MER), colistimethate or colistin (COL), and LIN. Moreover, in the present study, we examined each antibiotic’s independent potential contribution to the development of CDI. Consecutively, we evaluated data reported in EudraVigilance (EV), an extensive database for reporting adverse drug reactions (ADRs), as individual case safety reports (ICSRs). To evaluate the real-world situation, we compared the reports regarding CDI from both databases and related to patients’ exposure to all seven antibiotics.

## 2. Results

### 2.1. Descriptive Analysis of Reports from Sibiu County Clinical Emergency Hospital (SCCEH)

#### 2.1.1. Baseline Patients’ Characteristics

The patients’ characteristics are represented in Table 1. The average age of patients was 72.1 years. The most frequent cases were registered in the 65–85 years category (66.7%) and in the female group (55%). A favorable evolution was observed in 71.2% of patients. For surgical patients, favorable outcomes were registered for a high proportion of patients (86.8%) compared to non-surgical patients (63.0%). Regarding the detection mode, 57.7% of the cases were active, and 42.3% were passive.

#### 2.1.2. Hospital Length of Stay

**Figure 1:**
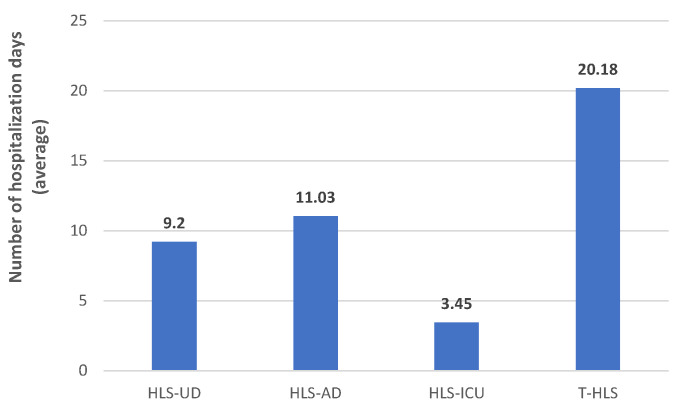
Hospital length of stay. HLS-AD—hospital length of stay after detection (days); HLS-ICU—hospital length of stay in ICU (days); HLS-UD—hospital length of stay until detection (days); T-HLS—total hospital length of stay (days).

In the studied group, the total hospital length of stay (T-HLS) was 20.18 days. The media of hospital length of stay until the detection (HLS-UD) was 9.2 days (minimum 0–maximum 34 days). A longer period was observed for the duration of hospitalization after CDI detection (11.03 days, minimum 0–maximum 31 days). The average hospital length of stay in ICU (HL-ICU) was 3.45 days (minimum 0–maximum 45 days) (Figure 1).

According to the data presented in Table 2, no statistical difference regarding the outcomes could be observed in the four categories (HLS-UD, HLS-AD, HLS-ICU, T-HLS).

#### 2.1.3. Influence of Age on the Patients’ Outcome

According to the data presented in Table 3, a favorable outcome was obtained in the first two subgroups (18–64 years and 65–85 years). As observed, the unfavorable outcome resulted in a proportion of 12% in the 18–64 years group, over 29.7% in the 65–85 years group, and 58.3% in people aged more than 85 years old.

#### 2.1.4. Wards

**Figure 2:**
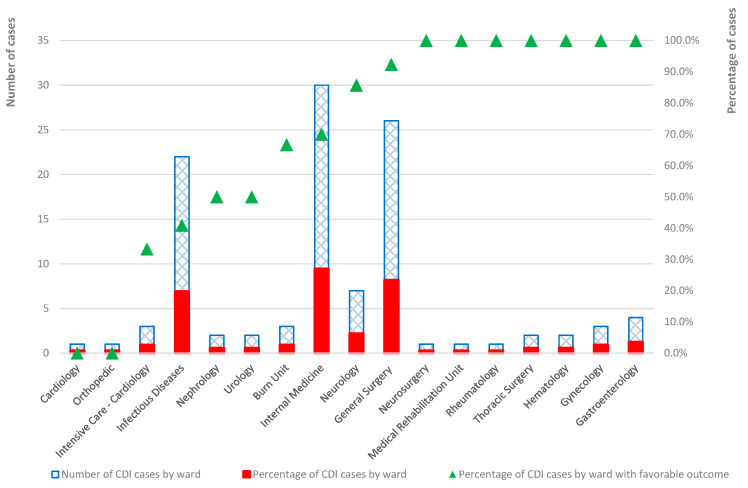
The distribution of cases by ward.

The highest number of cases was reported in internal medicine (n = 30, 27.0%), general surgery (n = 26, 23.4%), and infectious disease (n = 22, 19.8%). The rest of the wards reported fewer than four cases, except neurology (n = 7, 6.3%). In seven wards, the favorable outcomes represented 100% of total cases, and only in two wards, the favorable outcome represented 0%. It can be noticed that a higher percentage of favorable outcomes was reported in the wards with the most cases, such as general surgery (92.3%) and internal medicine (70.0%) (Figure 2).

#### 2.1.5. Admission Diagnosis

**Figure 3:**
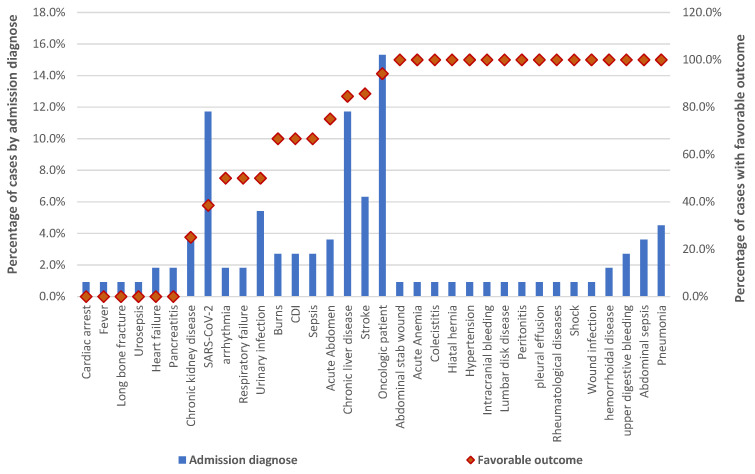
The distribution of cases by admission diagnoses. CDI—*Clostridioides difficile* infection.

According to the results presented in Figure 3, we observed that among the cases diagnosed with CDI in SCCEH, the majority of them were found in patients with an admission diagnosis of oncological pathology (n = 17, 15.3%; favorable outcome = 94.1%), SARS-COV2 (n = 13, 11.7%; favorable outcome = 38.5%), chronic liver disease (n = 13, 11.7%; favorable outcome = 84.6%), stroke (n = 7, 6.3%; favorable outcome = 85.7%), and urinary tract infection (n = 6, 5.4%; favorable outcome = 50%).

#### 2.1.6. Charlson Comorbidity Index

Table 4 presented the Charlson comorbidity index (CCI) for patients with CDI from SCCEH (average CCI: 7.6). No significant differences could be observed in females compared to males. Still, significant differences could be observed between age categories and evolution groups (favorable compared to unfavorable).

#### 2.1.7. Antibiotic Exposure

The mean of antibiotics used by all patients is 1.50 ± 1.09. In subgroup 1, the mean has a higher value (1.68 ± 1.22) than the other two subgroups (1.5 ± 1.11 for 65–85 years and 1.17 ± 0.39 for > 85 years subgroup). The median of the antibiotics used by all patients is 1. Except for subgroup 1 (median = 2), the other subgroups have a median equal to 1. The value of the mode (maximum repeated value) is 1 for the entire group and subgroups 2 and 3. For subgroup 1, the mode is 3 (Table 5).

Although there is no statistical difference regarding the outcomes, it can still be observed that the cases with favorable outcomes were exposed to a more significant number of antibiotics (1.53 ± 1.07) compared to the unfavorable ones (1.44 ± 1.13). The median of the antibiotics used by patients is 1 in both subgroups. (Table 6).

Regarding previous antibiotic exposure, the patients in the analyzed group were most frequently exposed to CFT (n = 32) and MER (n = 20) and the least to COL (n = 3). Exposure to the other four antibiotics was as follows: LIN (n = 11), GEN (n = 10), PIP/TAZ (n = 8), and CPX (n = 7).

**Figure 4:**
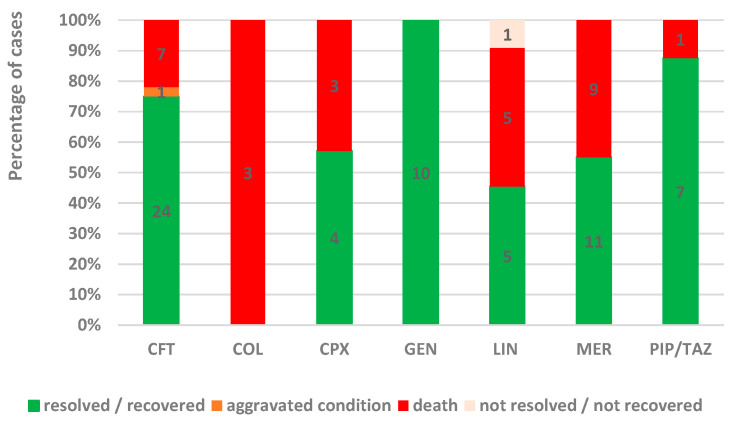
The percentage of cases by outcome in total reports. CFT—ceftriaxone; COL—colistin; CPX—ciprofloxacin; GEN—gentamicin; LIN—linezolid; MER—meropenem; PIP/TAZ—piperacillin and tazobactam.

According to Figure 4, the proportion of the recovered/resolved cases from the total cases was higher for GEN (100%, n = 10), PIP/TAZ (88%, n = 7), and CFT (75%, n = 24). Also, a lower proportion of recovered/resolved cases was registered in patients treated with COL (0%, n = 0), LIN (45%, n = 5), and MER (55%, n = 11). The highest proportion of fatal cases was registered for MER (45%, n = 9), LIN (45%, n = 5), and COL (100%, n = 3).

### 2.2. Analysis of Spontaneous Reports from EudraVigilance

#### 2.2.1. Descriptive Analysis of ICSRs Uploaded in 2022

In 2022, the EV database registered 249 ICSRs for all antibiotics analyzed in the present study, most of them being reported for CFT (n = 85), PIP/TAZ (n = 78), MER (n = 36), and CPX (n = 36). For all seven drugs analyzed, health professionals uploaded the majority of reports, but only for COL, GEN, and LIN, the majority of ICSRs were reported from the European Economic Area (EEA).

It can also be noted that CDI was most frequently reported for patients in the following age groups: 65–85 years (n = 107, 43.0%), more than 85 years (n = 67, 26.9%), and 18–64 years (n = 61, 24.5%).

The proportion of reports between the two genders was similar (male—122, female—124), but for males, CDI associated with the consumption of MER (72.2%), CFT (55.3%), and GEN (50%) were reported more frequently (Table 7).

#### 2.2.2. Outcomes

**Figure 5:**
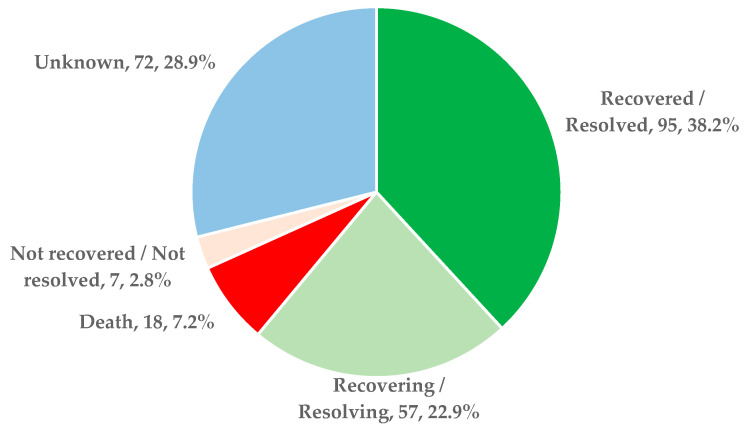
Outcomes presented in ICSRs reported in EV in 2022.

Figure 5 presents the outcomes included in ICSRs for all seven drugs analyzed. More than 61.1% of total reports are related to a favorable outcome (recovered/resolved—38.2% and recovering/resolving—22.9%). Death was reported in 18 ICSRs (7.2%), and not recovered/not resolved outcome was reported in 7 ICSRs (2.8%).

**Figure 6:**
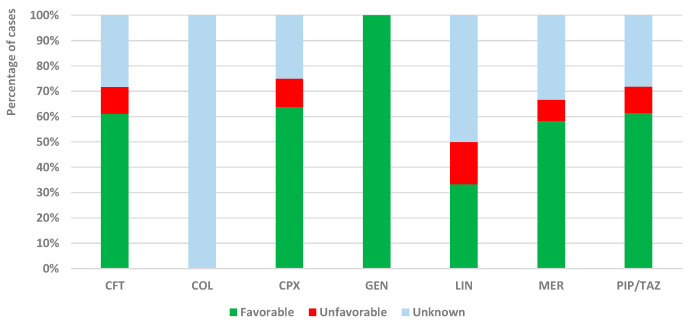
Distribution of ICSR by category of outcomes and antibiotic. CFT—ceftriaxone; COL—colistin; CPX—ciprofloxacin; GEN—gentamicin; LIN—linezolid; MER—meropenem; PIP/TAZ—piperacillin and tazobactam.

Although favorable results were reported for most antibiotics, unfavorable results were recorded in some reports for LIN (16.7%), CPX (11.1%), CFT (10.6%), and PIP/TAZ (10.3%) (Figure 6).

**Figure 7:**
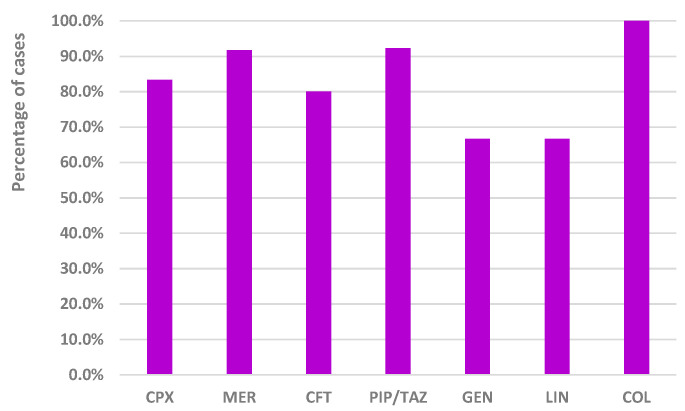
Distribution of cases associated with caused/prolonged hospitalization reported in EV in 2022. CFT—ceftriaxone; COL—colistin; CPX—ciprofloxacin; GEN—gentamicin; LIN—linezolid; MER—meropenem; PIP/TAZ—piperacillin and tazobactam.

Figure 7 showed that all analyzed antibiotics caused/prolonged hospitalization in percentages between 66.7% (GEN and LIN) and 100% (COL).

### 2.3. Comparison between the Reports from Sibiu County Clinical Emergency Hospital (SCCEH) and the Spontaneous Reports from EudraVigilance (EV)

#### 2.3.1. Exposure to Analyzed Drugs as a Single Suspected Antibiotic

**Figure 8:**
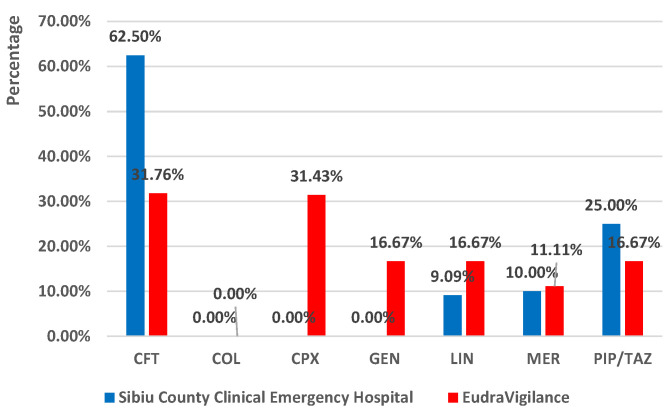
Comparison of the proportion of reports associated with each drug as the only suspected antibiotic in all reports associated with the analyzed drug in SCCEH vs. EudraVigilance (2022). CFT—ceftriaxone; COL—colistin; CPX—ciprofloxacin; GEN—gentamicin; LIN—linezolid; MER—meropenem; PIP/TAZ—piperacillin and tazobactam.

Figure 8 showed a similar situation in SCCEH and EV, referring to the proportion of cases associated with COL (0%—SCCEH, 0%—EV) and MER (11.11%—EV, 10%—SCCEH) as a single suspected antibiotic for CDI. A large difference was noticed regarding the CPX (0%—SCCEH, 31.43%—EV), CFT (62.5%—SCCEH, 31.76%—EV), and GEN (0%—SCCEH, 16.67%—EV) as being the only suspected antibiotics.

#### 2.3.2. Frequency of Exposure to Other Antibiotics in Cases Where the Analyzed Drug Was Not the Only Suspected Antibiotic

**Figure 9:**
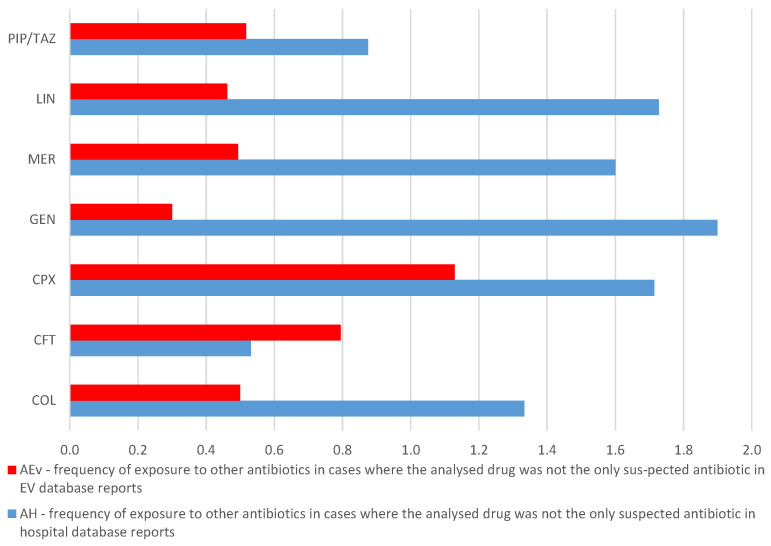
Frequency of exposure to other antibiotics in cases where the analyzed drug was not the only suspected antibiotic—comparison between A_Ev_ and A_H_ reports. CFT—ceftriaxone; COL—colistin; CPX—ciprofloxacin; GEN—gentamicin; LIN—linezolid; MER—meropenem; PIP/TAZ—piperacillin and tazobactam.

The frequency of exposure to other antibiotics in cases where the analyzed drug was not the only suspected antibiotic in EV database reports (A_Ev_) and in hospital database reports (A_H_), respectively, are presented in Figure 9. Similar values could be observed for CFT (A_Ev_ = 0.8 and A_H_ = 0.5) and PIP/TAZ (A_Ev_ = 0.5 and A_H_ = 0.9) (Figure 9).

**Figure 10:**
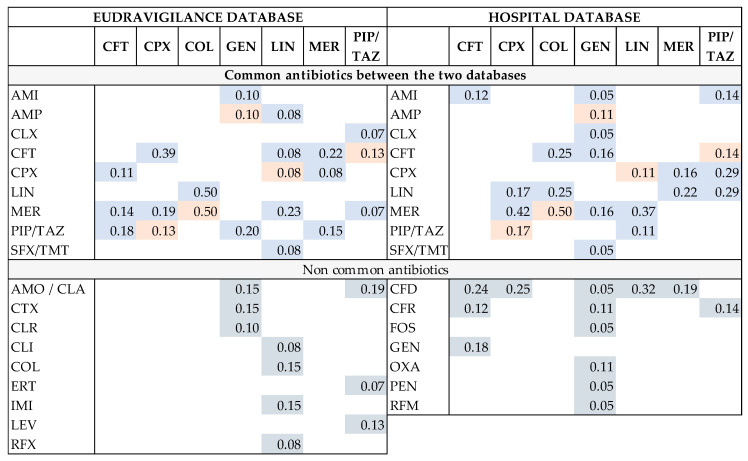
Comparison between the most frequently reported antibiotics as suspected drugs in CDI cases associated with the analyzed antibiotics in EudraVigilance and SCCEH database in 2022. The numbers represent the frequency of exposure (R) to each suspected antibiotic, in the total number of exposures for each of the seven studied antibiotics. AMI—amikacin; AMO/CLA—amoxicillin + clavulanic acid; AMP—ampicillin; CFD—ceftazidime; CFR—cefuroxime; CFT—ceftriaxone; CLR—clarithromycin; CLX—cephalexin; COL—colistin; CPX—ciprofloxacin; CTX—cefotaxime; ERT—ertapenem; FOS –fosfomycin; GEN—gentamicin; IMI—imipenem + cilastatin; LEV—levofloxacin; LIN—linezolid; MER—meropenem; MOX—moxifloxacin; OXA—oxacillin; PEN—penicillin G; RFM—rifampicin; PIP/TAZ—piperacillin and tazobactam; RFX—rifaximin. The red cells include results with similar R values; the blue cells include results with non-similar R values; and the grey cells include results that can not be compared.

Subsequently, we identified the other suspected antibiotics associated with the analyzed drug. The frequency of exposure (R) to each suspected antibiotic, in the total number of exposures for each of the seven studied antibiotics, was compared between the two databases. Thus, in Figure 10, it can be observed that amoxicillin/clavulanic acid was most frequently reported as a suspected drug in CDI cases associated with GEN and PIP/TAZ in the EV database but not found in any SCCEH reports. The proportion of reports showing ampicillin as a suspected drug in CDI cases associated with GEN is similar in both databases (SCCEH: R = 0.10 and EV: R = 0.11). The same observation is available for MER in CDI cases associated with COL (SCCEH: R = 0.50 and EV: R = 0.50), CFT in CDI cases associated with PIP/TAZ (SCCEH: R = 0.14 and EV: R = 0.13), and PIP/TAZ in CDI cases associated with CPX (SCCEH: R = 0.17 and EV: R = 0.13).

## 3. Discussion

CDI is a frequently occurring disease that affects patients previously exposed to antibiotics. This infection is an essential concern for healthcare professionals, as it can lead to significant morbidity and mortality worldwide [23].

From 30,608 patients admitted to SCCEH in 2022, 111 patients were diagnosed with CDI, representing an incidence rate of 3.63 per 1000 admissions. A study performed in another hospital in Romania revealed a high incidence of CDI (20.57/15.70 to 1000 discharged patients in 2013/2014) [24]. A meta-analysis that included 229 publications with data from 41 countries identified a yearly incidence of up to 35.15 CDI per 1000 admissions [25]. A retrospective, multicenter cohort study performed in 43 hospitals in the United States of America between 1 January 2013 and 31 December 2017 showed that the median total incidence has increased from 7.9 CDIs per 1000 admissions to 9.3 CDIs per 1000 admissions [26]. Also, a study by Kuntz et al. reported a higher incidence (13.3 CDIs per 1000 patient admissions) [27] than that observed in SCCEH. On the other hand, a teaching hospital from Okayama City (Japan) reported a low incidence rate, between 1.71 cases per 1000 admissions in an old hospital and 0.46 cases per 1000 admissions in a new hospital [28]. Also, a low incidence of CDI was recorded in a Portuguese hospital (20.7 per 10,000 admissions) [29]. The mortality rate from SCCEH was 252.3 deaths/1000 cases/year (25%). Mortality rates due to CDI vary widely between studies. A systematic review of HA-CDI in Europe based on studies published between 2000 and 2010 estimated in-hospital mortality ranging between 0% (Latvia) and 44% (Austria) [30]. A retrospective study of patients diagnosed with CDI in a healthcare facility in Taiwan reported an in-hospital mortality of 28.7% [31]. Another multicenter cohort study from the Netherlands found a 2.5-fold increase in 30-day mortality due to CDI [32]. Age and the presence of comorbidities were found to be among the most reported risk factors for mortality in CDI patients [33].

A significant proportion (77.5%) of all patients admitted to SCCEH and affected by CDI are represented by the elderly population (≥65 years). Also, in the three age subgroups, the proportion of unfavorable outcomes doubles from one group to another. Thus, elderly people who are over 85 years old are exposed to a higher risk of unfavorable outcomes associated with CDI. Also, in the descriptive analysis of the spontaneous reports recorded in EV during 2022 and associated with the use of CFT, COL, CPX, GEN, LIN, MER, and PIP/TAZ, it was observed that CDI was most frequently reported in patients aged 65–85 years, followed by individuals over 85 years. According to other studies, the elderly have a weakened immune response, which makes them more vulnerable to infections, including CDI, especially when they have other illnesses [34,35]. The elderly population is often exposed to long-term medical care and interventions, and frequent drugs used by this patient population (e.g., antibiotics and proton pump inhibitors) can disrupt the microbial balance, creating an environment for CD proliferation [35,36,37,38]. Moreover, according to the literature, the mortality rate within 30 days is higher in individuals over the age of 60. This risk increases substantially in those aged 80 and above. This highlights the increased susceptibility of older populations, particularly those over 80, to the negative consequences of CDI [39].

In total, 55% of all patients admitted to SCCEH and affected by CDI were females. Regarding the occurrence of CDI-related ADRs recorded in EV, no major difference was observed between females (50.4%) and males (49.6%). Previous studies have shown that females have higher rates of CDI compared to males [40,41]. This suggests that sex-specific dynamics, particularly concerning the gut microbiome, may be at play. Research has revealed that differences in the gut microbiome between males and females are closely linked to hormonal variations. Hormone levels have been identified as mediators influencing the distinct microbial composition observed in both sexes [42,43].

Out of all the cases of CDI from SCCEH, only 34.2% were surgical cases. However, we observed that 86.8% of these cases had favorable outcomes, suggesting good antibiotic stewardship in terms of antibiotic prophylaxis. On the other hand, non-surgical patients had favorable outcomes in only 63% of cases. This indicates a significant difference in the clinical paths and ultimate prognosis between these two groups of patients. Several studies and reports have shown a strong link between the occurrence of CDI in individuals who undergo surgical procedures [44,45]. This correlation is mainly attributed to the widespread use of broad-spectrum antibiotics in surgical care. Moreover, the increasing incidence of CDI in surgical patients is closely related to the rising number of elderly individuals and those with weakened immune systems who undergo various surgical interventions [44]. A recent study found that patients undergoing surgical procedures are more likely to develop severe CDI [46]. Although these patients tend to have a more challenging clinical course with CDI, their overall outcomes are better than those of medical patients [46]. On the other hand, medical patients experience a shorter hospital stay, an earlier onset of CDI, and higher rates of 30-day and overall mortality, with deaths occurring earlier after the onset of CDI [46].

The results of our study showed that, on average, it takes around 9.2 days from the time of admission until the detection of CDI in hospitalized patients from SCCEH. Additionally, once CDI is identified, the average hospital stay significantly increases to 11.03 days. However, the reports from EV showed that COL, PIP/TAZ, MER, CFT, and CPX were associated with caused/prolonged hospitalization in a high proportion (100%, 92.3%, 91.7%, 80%, and 83.3%, respectively). A recent study found that CDI adds an average of three days to hospital stays, significantly impacting hospital discharge rates [47]. For example, in the context of the United Kingdom, CDI was linked to a considerable reduction in the daily discharge rate, specifically by about 28% [48]. On the other hand, a prospective study has reported a relatively short duration of hospitalization, with a median stay of only five days and a range spanning from 3 to 11 days [49]. The difference in hospitalization duration highlights the variability across different studies and the importance of considering contextual factors and study design when interpreting such results.

The highest incidence of cases reported across various medical and surgical wards from SCCEH was found in infectious disease (n = 22, 19.8%), general surgery (n = 26, 23.4%), and internal medicine (n = 30, 27.0%). A noteworthy trend emerged when examining outcomes. In seven wards, favorable outcomes represented 100% of total cases, highlighting a notable success in patient management. On the other hand, out of all the wards, only two (cardiology and orthopedics) had a 0% rate of favorable outcomes, indicating difficulties in achieving positive patient results in those particular contexts. However, it is essential to consider that the patients in those wards were of advanced age and had severe underlying medical conditions and a high comorbidity index. According to a recent study, the ICU and internal medicine wards have a higher prevalence of CDI cases. The analysis revealed that the median number of CDI cases per admission was consistently higher in these wards and had the highest incidence rate and density [25]. Another recent study showed a notable number of CDI cases in general medicine wards. The study further suggests that patients admitted to general medicine wards tend to be older and may have pre-existing medical conditions that make them more susceptible to acquiring the infection [50].

The Charlson comorbidity index (CCI) is reliable for assessing critical outcomes such as mortality, hospital stay, functional disability, and healthcare utilization [51]. Patients with a high CCI score, mainly those exceeding 7, are more likely to experience recurrent condition occurrences [52,53]. In our study, we analyzed the application of the CCI for patients from SCCEH diagnosed with CDI. The average CCI of the patients was calculated at 7.6. This indicates a significant burden of comorbidities that the studied patient population had. While analyzing the data by gender, we observed no significant differences between men and women regarding the CCI scores. However, a pattern could be observed when exploring the correlation between CCI, age categories, and the evolution of CDI outcomes. Some significant differences were identified, particularly when comparing different age groups within the favorable and unfavorable evolution categories. These differences underscore the importance of considering demographic and comorbidity factors in managing CDI.

Specific diagnoses have a high incidence of unfavorable outcomes, as shown in our analysis. In some instances, the gravity of concurrent diagnoses leads to 100% unfavorable outcomes. This includes specific conditions such as cardiac arrest (n = 1), fever (n = 1), long bone fracture (n = 1), urosepsis (n = 1), heart failure (n = 2), and pancreatitis (n = 2), underlining the critical nature of these medical situations. In total, 75% of cases with chronic kidney disease (n = 4) had unfavorable outcomes. This substantial proportion emphasizes the challenging clinical trajectory associated with chronic kidney disease. The impact of SARS-CoV-2 is particularly noteworthy, with 61.5% of cases (n = 13) leading to unfavorable outcomes. This underscores the complex and often unpredictable nature of outcomes associated with COVID-19. Additionally, diagnoses such as arrhythmia (n = 2), respiratory failure (n = 2), and urinary infection (n = 3) exhibit a 50% frequency of unfavorable outcomes.

Regarding the exposure of patients to antibiotics, it was observed that in SCCEH, the mean of antibiotics used by all patients is 1.50. According to the United States Centers for Disease and Control Prevention, exposure to antibiotics increases the risk of CDI by 7 to 10 times by killing the good germs from saprophytic flora capable of fighting against opportunistic bacteria like CD [54,55]. A recent study examined the effect of prior antibiotic use on the risk of developing CDI. The study found that the amount of antibiotics used before hospitalization is the most significant factor contributing to the risk of developing CDI [56]. Additionally, the study suggests that while all types of antibiotics carry some risks, the level of risk varies depending on the specific drug and method of administration [56]. The results are consistent with previous studies, indicating that the risk of developing CDI is higher in individuals exposed to antibiotics. The risk of developing CDI is cumulative and increases with each day of antibiotic exposure [57]. The most significant risk occurs within the first 60 days after taking antibiotics. In the dataset analyzed, the odds of developing CDI increased by 12.8% per day of individual antibiotic exposure [58,59].

Moreover, the elderly people (>85 years) were exposed only to 1.17 antibiotics compared to all groups (1.50). Statistical analysis shows that the coefficient of skewness has positive values for the entire group (0.66) and subgroups 2 (0.73) and 3 (2.06). For subgroup 1, a symmetrical distribution of values (skewness = 0.07) could be observed, and a constant trend in the use of antibiotics could be implicitly observed. Additionally, a leptokurtic distribution (kurtosis > 0) could be noticed in all group, subgroups 2, and 3, showing a small range, variance, and standard deviation with most data points near the mean. The opposite of subgroup 1 could be observed as a platykurtic distribution (kurtosis < 0), which shows an extensive range, variance, and standard deviation [60,61]. Moreover, patients who had favorable outcomes were exposed to a slightly higher number of antibiotics (1.53) compared to those who had unfavorable outcomes (1.44). Skewness has positive values for both subgroups (0.65 and 0.73). These values indicate asymmetry [60,61] and could suggest a mildly increasing tendency to use antibiotics. Additionally, a leptokurtic distribution (kurtosis > 0) could be noticed in patients from the subgroup with favorable outcomes (kurtosis = 0.439452) that shows a small range, variance, and standard deviation with the majority of data points near the mean. In subgroups with unfavorable outcomes, a normal distribution (kurtosis = −0.07617) [60,61] is observed.

Of the seven studied antibiotics, patients from SCCEH were most frequently exposed to CFT and MER. In the EV database, most CDI-related ADRs were reported for CFT, PIP/TAZ, MER, and CPX. Moreover, our results indicated that there are some similarities between cases associated with COL and MER between the two databases regarding the patient exposure to each analyzed drug as a single suspected antibiotic, while there were large differences between cases associated with CFT and CPX. Furthermore, for all analyzed antibiotics except CFT, exposure to other antibiotics was increased in SCCEH reports compared to EV. Some antibiotics were frequently reported as suspected drugs only in the EV database: (i) AMO/CLA, CTX, and CLR in CDI cases associated with GEN; (ii) CLI, COL, IMI, and RFX in CDI cases associated with LIN; (iii) AMO/CLA, ERT, and LEV CDI cases associated with PIP/TAZ. Only in the reports from SCCEH, it was observed that (i) CFD was frequently reported as a suspected drug in CDI cases associated with CFT, CPX, GEN, LIN, and MER; (ii) CFR in CDI cases associated with CFT, GEN, and PIP/TAZ; and (iii) FOS, OXA, PEN, RFM in CDI cases associated with GEN. A recent study compared the risk of CDI associated with 27 antibiotics. The results showed that clindamycin has a high risk of causing CDI. The fluoroquinolones, such as ciprofloxacin and moxifloxacin, as well as later-generation cephalosporin like cefaclor, have odds ratios ranging from 4.16 to 6.83. Meanwhile, linezolid, cefprozil, cephalexin, cefadroxil, ampicillin, levofloxacin, and sulfamethoxazole/trimethoprim have odds ratios between 2.15 and 3.58 [62]. A hospital-acquired CDI meta-analysis found that certain antibiotics are more strongly associated with CDI than others. The order of association from strongest to weakest is third-generation cephalosporins, clindamycin, second-generation cephalosporins, fourth-generation cephalosporins, carbapenems, trimethoprim-sulfonamides, fluoroquinolones, and penicillin combinations [63].

To ensure the safety of patients in hospitals, it is important to implement strict infection control measures and sanitation protocols to address bacterial contamination. The issue of CD contamination in hospitals can be effectively tackled through a comprehensive approach that includes multiple strategies. These strategies may include promoting the responsible use of antibiotics, implementing effective infection control practices, enhancing environmental cleaning, providing education and training on infection prevention, using probiotics, adopting early detection methods, and isolating affected patients [64].

### Limitations

As this is a retrospective observational study, it has some limitations. The level of evidence is low, and this study’s representativeness is limited due to inconsistent data reporting, uneven use of terminology, etc. This study’s small representation in the general population is due to data being reported in a year and collected only from a single center. The lack of clinical and laboratory information can cause a possible alteration in the identification of the first symptoms to establish the onset of the disease and the identification of all cases of CDI, which could lead to an under-reporting of these cases. Not all risk factors have been identified, so causality cannot be established. The outcomes could be potentially influenced by differences between the specialists’ vigilance in detecting non-severe cases of CDI. It should be noted that this study did not include all of the cumulative risk factors for CDI. Some factors that were not considered include using proton pump inhibitors or anti-inflammatory medications, prolonged hospitalization exceeding 20 days, surgical interventions on the digestive tract, the presence of a nasogastric tube, or parenteral nutrition. The patients’ follow-up could be considered another study limitation due to the lack of information regarding their evolution after discharge, especially in patients with improved or aggravated status.

Although our study has significant advantages based on the EV database data extraction technology, there are also limitations that we need to consider. First of all, the EV database is a spontaneous reporting system. This means the reporting may be selective, incomplete, inaccurate, and unverified. Thus, it is not easy to take into account certain factors such as dose, duration of use, comorbidities, drug combinations, and other factors that may influence the occurrence of CDI. Secondly, the EV database only contains cases with adverse events, and the incidence rate cannot be calculated due to the lack of the total number of patients receiving antibiotic treatment. In other words, we do not have the denominator of drug exposure. Finally, disproportionality analysis based on EV did not quantify risk or causality but only assessed signal strength. Therefore, it is essential to remember that the analysis only indicates a signal that needs further investigation but does not provide conclusive evidence.

One issue with spontaneous reporting is the occurrence of duplicate reports, where multiple sources submit the same report (a patient and a medical professional). Additionally, there may be various reports where the reporter modifies an existing case follow-up report with additional information. To address this, EV periodically identifies and merges these duplicate reports during quality review. Furthermore, in this study, we used a deduplication procedure to identify and eliminate these reports based on the unique EU local number code, making the reporting process more efficient and accurate. The two databases can only be compared in a few aspects due to the differing levels and information types. The case reports in the two databases differ, making it difficult to compare the results.

## 4. Materials and Methods

### 4.1. Study Design

A retrospective pharmacovigilance study referring to CDI was performed. The real-world data reported in 2022 from SCCEH were analyzed. All cases investigated (n = 111) from the hospital’s database were classified as nosocomial infections associated with medical procedures, with none attributed to community transmission. The infections observed in SCCEH are classified by the hospital’s infection surveillance department as nosocomial infections. Nosocomial CDI was defined as the development of new-onset diarrhea either at admission in patients with recent hospitalization within twelve weeks or ≥48 h from admission in patients without recent prior hospitalization, plus a confirmed CDI [65]. Institutional review board approval was obtained before the initiation of this study.

Subsequently, another study, including a descriptive analysis, was performed based on the spontaneous reports registered in the EV database (n = 249) between 1 January and 31 December 2022 at https://www.adrreports.eu (accessed on 11 October 2023) [66]. The ICSRs refer to the EEA or non-EEA and could be reported by healthcare professionals or non-healthcare professionals (e.g., patients, lawyers, etc.) [67]. For this study, no ethics committee approval is required because ICSRs do not include any patients’ personal information [68].

### 4.2. Materials

Seven antibiotics that are frequently used in hospital settings were chosen: CFT, COL, CPX, GEN, LIN, MER, and PIP/TAZ. The analyzed data were reported in EV between 1 January and 31 December 2022. Preferred terms related to CDI were as follows: “Clostridial infection”, “Clostridial sepsis”, “*Clostridium* bacteremia”, “*Clostridium* colitis”, “*Clostridium difficile* colitis”, “*Clostridium difficile* infection”, and “Gastroenteritis clostridial”.

### 4.3. Data Analysis

#### 4.3.1. Descriptive Analysis of Reports from Sibiu County Clinical Emergency Hospital

A descriptive analysis of data registered in 2022 in SCCEH was realized. Many criteria were considered to evaluate the baseline patients’ characteristics:

- Demographic data: age, gender, patient’s category (surgical or non-surgical patient). The data collected from SCCEH referred to adult patients, and the age categories were chosen according to European Medicines Agency regulations regarding pharmacovigilance activity. This is to enable better comparison between the two datasets.
- Evolution: resolved/recovered, aggravated conditions, not resolved/not recovered (transfers), or death. The resolved/recovered cases represented the favorable evolution, and unfavorable evolution included aggravated conditions, not resolved/not recovered (transfers included), or death.
- Type of detection: active or passive. The active or passive detection mode indicates how the infection was reported to the infection surveillance department from SCCEH. Active detection means that the patient’s attending physician reported the infection, while passive detection means that the infection surveillance department detected the infection.

The descriptive analysis presents (i) the influence of age on the outcome (favorable or unfavorable), (ii) the distribution of cases by wards (cases, percentage of total cases, proportion of favorable outcomes), (iii) the distribution of cases by medical diagnoses (cases, percentage of total cases, proportion of favorable outcomes), (iv) CCI (for the group, by sex, by age category, by outcome), (v) hospital length of stay (HLS-AD—hospital length of stay after the detection, HLS-ICU—hospital length of stay in ICU, HLS-UD—hospital length of stay until the detection, and T-HLS—total hospital length of stay), and (vi) exposure to antibiotics (analysis regarding the antibiotics’ exposure, distribution of cases by the number of antibiotics used and outcome, the proportion of cases by outcome in relation with the total number of cases). All administered antibiotics in the last month were considered suspected except those specific for treating CDI (vancomycin, metronidazole, tigecycline, and rifaximin). The value of CCI was used to predict the 10-year survival in patients with multiple comorbidities. This indicator was calculated with MDcalc [69], and the values took into account comorbidities such as diabetes, cancers, cardiovascular diseases, renal failure, AIDS, etc. [70].

#### 4.3.2. Analysis of Spontaneous Reports from EudraVigilance

A descriptive analysis of CDI reported as a spontaneous adverse reaction related to using CFT, COL, CPX, GEN, LIN, MER, and PIP/TAZ was performed. Many criteria were used to carry out this analysis: age category, gender, reporter group, and outcomes. The resolved/recovered cases and resolving/recovering were considered with a favorable evolution, and an unfavorable evolution was considered for not resolved/not recovered or death conditions.

#### 4.3.3. Comparison between the Reports from Sibiu County Clinical Emergency Hospital (SCCEH) and the Spontaneous Reports from EudraVigilance (EV)

To carry out a comparison between both databases, the proportion of reports associated with each drug as the only suspected antibiotic in all reports associated with the analyzed drug in SCCEH versus EudraVigilance was determined.

Moreover, the frequency of exposure to other antibiotics in cases where the analyzed drug was not the only suspected antibiotic in EV database reports (A_Ev_) and in hospital database reports (A_H_), respectively, was examined. A_Ev_ was calculated as the ratio of the total number of other antibiotics suspected of CDI reported in association with each analyzed drug (COL, CFT, CPX, GEN, LIN, MER, or PIP/TAZ) to the total number of reports registered for each analyzed drug. A similar ratio was calculated for hospital settings (A_H_). Consecutively, both ratios (A_Ev_ and A_H_) were compared.

(1) $$A_{\mathrm{Ev}}=\frac{N_{\mathrm{SEV}}}{N_{\mathrm{EV}}}$$

A_Ev_ = frequency of exposure to other antibiotics in cases where the analyzed drug was not the only suspected antibiotic in EV database reports;

N_SEV_ = total number of other antibiotics suspected of CDI reported in association with each analyzed drug in EV database reports;

N_EV_ = total number of reports registered for each analyzed drug in the EV database.

(2) $$A_{H}=\frac{N_{\mathrm{SH}}}{N_{H}}$$

A_H_ = frequency of exposure to other antibiotics in cases where the analyzed drug was not the only suspected antibiotic in hospital database reports;

N_SH_ = total number of other antibiotics suspected of CDI reported in association with each analyzed drug in hospital database reports;

N_H_ = total number of reports registered for each analyzed drug in the hospital database.

Subsequently, we identified the other suspected antibiotics associated with the analyzed drug. The frequency of exposure to each suspected antibiotic, in the total number of exposures for each of the seven studied antibiotics, was compared between the two databases.

A frequency indicator (R) was obtained for both databases (hospital and EudraVigilance) as the ratio of the number of reports for each other suspected antibiotic identified (N_A_) and the total number of other suspected antibiotics associated with each studied drug (N_TA_). The first three frequency values for both databases (hospital and EV) were extracted and used for comparison.

(3) $$R=\frac{N_{A}}{N_{\mathrm{TA}}}$$

where:

R—frequency indicator;

N_A_—number of reports for each other suspected antibiotic identified;

N_TA_—total number of other suspected antibiotics associated with each studied drug.

### 4.4. Statistical Analysis

The data were analyzed using Microsoft Excel 2010 software—Data Analysis Tools. The variables that describe the characteristics of the population were presented in absolute numbers, frequencies, and percentages, or mean and standard deviation. To evaluate the antibiotics exposure, skewness (a measure of symmetry) and Kurtosis (a measure that quantifies the shape of the probability distribution) were considered. The comparison between subgroups was considered significant if *p*-value < 0.05.

## 5. Conclusions

This study stands out by using a unique approach, comparing the results from two different datasets collected from a clinical setting and the European spontaneous reporting system. This methodology helps us to better understand the clinical characteristics and the effects of antibiotic use on CDI, providing a more accurate representation of real-world outcomes. The present study offers valuable insights to the scientific community, emphasizing the crucial need for responsible antibiotic use and effective infection prevention and control measures. Future studies are encouraged to investigate further into the complexities of antibiotic-associated colitis, to enhance our knowledge and improve signal detection in pharmacovigilance practices.

## Figures and Tables

**Table 1:** *Clostridium difficile* infection patients’ characteristics.

	Patient Characteristic	Cases n (%)
Age Mean *(Minimum–Maximum)*	72.1 years *(18.3–94.8 years)*	111
Age category * Mean *(Minimum–Maximum)*	18–64 years 54.7 years *(18.3–63.9 years)*	25 (22.5)
	65–85 years 75.1 years *(65.3–84.3 years)*	74 (66.7)
	>85 years 89.7 years *(85.8–94.8 years)*	12 (10.8)
Gender	Female	61 (55.0)
	Male	50 (45.0)
Patients’ category	Surgical	38 (34.2)
	favorable outcome	33 (86.8)
	unfavorable outcome	5 (13.2)
	Non-surgical	73 (65.8)
	favorable outcome	46 (63.0)
	unfavorable outcome	27 (37.0)
Evolution	Favorable	79 (71.2)
	resolved/recovered	79 (100)
	Unfavorable	32 (28.8)
	aggravated condition	3 (9.0)
	Death	28 (88.0)
	not resolved/not recovered	1 (3.0)
Detection mode	Active	64 (57.7)
	Passive	47 (42.3)

* *p* < 0.001.

**Table 2:** The relationship between the number of hospitalization days and patients’ outcomes. HLS-AD—hospital length of stay after detection (days); HLS-ICU—hospital length of stay in ICU (days); HLS-UD—hospital length of stay until detection (days); T-HLS—total hospital length of stay (days).

	Outcome	Average Duration of Hospitalization (Days)	*p*-Value
HLS-UD	favorable	9.78	*p* > 0.05
	unfavorable	7.75	
HLS-AD	favorable	11.08	*p* > 0.05
	unfavorable	10.91	
HLS-ICU	favorable	2.57	*p* > 0.05
	unfavorable	5.63	
T-HLS	favorable	20.80	*p* > 0.05
	unfavorable	18.66	

**Table 3:** The distribution of the outcome by age category. n—number of patients.

Age Category	Favorable n (%)	Unfavorable n (%)
18–64 years	22 (88.0)	3 (12.0)
65–85 years	52 (70.3)	22 (29.7)
>85 years	5 (41.7)	7 (58.3)

**Table 4:** Charlson comorbidity index for patients with *Clostridoides difficile* infection.

Variables		Charlson Comorbidity Index		*p*-Value
		Average	Range	
All patients		7.6	(0–16 points)	
Gender	Female	8.04	(4–15 points)	*p* > 0.05
	Male	7.14	(0–16 points)	
Category of age	18–64 years	6	(4–16 points)	*p* < 0.01
	65–85 years	8.1	(0–15 points)	
	>85 years	8.2	(6–11 points)	
Outcome	Favorable	7.2	(0–15 points)	*p* < 0.01
	*resolved/recovered*	*7.2*	*(0–15 points)*	
	Unfavorable	8.8	(4–16 points)	
	*aggravated condition*	*8.3*	*(7–11 points)*	
	*death*	*8.8*	*(4–16 points)*	
	*not resolved/not recovered*	*11*	*(11 points)*	

**Table 5:** The exposure of patients to antibiotics.

	All Group	Subgroup 1	Subgroup 2	Subgroup 3
		18–64 Years	65–85 Years	>85 Years
Mean	1.504505	1.68	1.5	1.166667
Standard Error	0.103084	0.243036	0.129434	0.112367
Median	1	2	1	1
Mode	1	3	1	1
Standard Deviation	1.086059	1.215182	1.11343	0.389249
Sample Variance	1.179525	1.476667	1.239726	0.151515
Kurtosis	0.202974	−1.16656	0.517144	2.64
Skewness	0.66032	0.07172	0.734379	2.055237
Range	5	4	5	1
Minimum	0	0	0	1
Maximum	5	4	5	2
Sum	167	42	111	14
Count	111	25	74	12

**Table 6:** Distribution of cases by number of antibiotics and outcome.

	Favorable	Unfavorable
Mean	1.531646	1.4375
Standard Error	0.120633	0.200491
Median	1	1
Mode	1	1
Standard Deviation	1.072206	1.134147
Sample Variance	1.149627	1.28629
Kurtosis	0.439452	−0.07617
Skewness	0.652101	0.730805
Range	5	4
Minimum	0	0
Maximum	5	4
Sum	121	46
Count	79	32

**Table 7:** Characteristics of ICSR related to CDI recorded in EudraVigilance (1 January–31 December 2022). EEA—European Economic Area, non-EEA—non-European Economic Area; HP—healthcare professionals, N-HPs—non-healthcare professionals; n—number of reports.

			CFT	COL	CPX	GEN	LIN	MER	PIP/TAZ
Total ICSRs, n			85	2	36	6	6	36	78
Reporter Group	HPs	n	85	2	30	6	6	34	76
		(%)	(100)	(100)	(85.7)	(100)	(100)	(94.4)	(97.4)
	N-HPs	n	0	0	5	0	0	2	2
		(%)	(0.0)	(0.0)	(14.3)	(0.0)	(0.0)	(5.6)	(2.6)
Countries	EEA	n	27	2	7	6	5	10	14
		(%)	(31.8)	(100.0)	(20.0)	(100.0)	(83.3)	(27.8)	(17.9)
	Non-EEA	n	58	0	28	0	1	26	64
		(%)	(68.2)	(0.0)	80.0)	(0.0)	(16.7)	(72.2)	(82.1)
Age Category	2 months–2 years	n	0	0	0	0	0	0	2
		(%)	(0.0)	(0.0)	(0.0)	(0.0)	(0.0)	(0.0)	(2.6)
	3–11 years	n	1	0	0	0	0	0	1
		(%)	(1.2)	(0.0)	(0.0)	(0.0)	(0.0)	(0.0)	(1.3)
	18–64 years	n	17	2	13	1	2	13	13
		(%)	(20.0)	(100.)	(36.1)	(16.7)	(33.3)	(36.1)	(16.7)
	65–85 years	n	34	0	17	3	4	18	31
		(%)	(40.0)	(0.0)	(47.2)	(50.0)	(66.7)	(50.0)	(39.7)
	>85 years	n	31	0	4	1	0	4	27
		(%)	(36.5)	(0.0)	(11.1)	(16.7)	(0.0)	(11.1)	(34.6)
	Not specified	n	2	0	2	1	0	1	4
		(%)	(2.4)	(0.0)	(5.6)	(16.7)	(0.0)	(2.8)	(5.1)
Gender	Male	n	47	0	14	3	2	26	30
		(%)	(55.3)	(0.0)	(38.9)	(50.0)	(33.3)	(72.2)	(38.5)
	Female	n	38	2	22	3	4	9	46.00
		(%)	(44.7)	(100.0)	(61.1)	(50.0)	(66.7)	(25.0)	(59.0)
	Not specified	n	0.00	0.00	0.00	0.00	0.00	1.00	2.00
		(%)	(0.0)	(0.0)	(0.0)	(0.0)	(0.0)	(2.8)	(2.6)

## Data Availability

Data are contained within this article.
